# Supplementary material for: MetaRibo-Seq measures translation in microbiomes
Source: Nat Commun. 2020 Jun 29;11:3268. doi: 10.1038/s41467-020-17081-z (PMC7324362; doi:10.1038/s41467-020-17081-z)
Supplement: Supplementary file 10 — Supplementary Data 7 [file 41467_2020_17081_MOESM10_ESM.zip › File2/Confidence_High_Taxonomy/83728_out.krona.html]

Javascript must be enabled to view this page.

members
magnitude
magnitudeUnassigned
count
unassigned
taxon
rank

83728\_out

3


SRS143466\_contig\_number\_contig-100\_9637.134457
1

2
2
superkingdom

976
phylum
1

class
117743
1

order
200644
1

49546
family
1

1
genus
111500

1
species
475082

SRS012273\_contig\_number\_contig-100\_13289.44236

1
1239
phylum

1
class
186801

186802
order
1


SRS014923\_contig\_number\_36084
species
172733
1
